# Emergence of carbapenemase-producing Enterobacterales (CPE) in patients with severe COVID-19 infection and successful control in intensive care

**DOI:** 10.1017/ash.2024.373

**Published:** 2024-10-31

**Authors:** Anthony Longhitano, Duncan Campbell, Nomvuyo Mothobi, Alison McKenzie, Caroline Bartolo, Sajal Saha, Norelle Sherry, Eugene Athan

**Affiliations:** 1 Department of Infectious Diseases, Barwon Health, Geelong, VI, Australia; 2 Department of Microbiology, Australian Clinical Labs, Geelong, VI, Australia; 3 Infection Prevention & Control, Barwon Health, Geelong, VI, Australia; 4 Deakin University, Geelong, VI, Australia; 5 MDU University of Melbourne, Parkville, VI, Australia; 6 Austin Health, Heidelberg, VI, Australia

## Abstract

**Objective:**

We aim to highlight the risks of acquiring carbapenemase-producing Enterobacterales (CPE) resistance genes in patients with severe coronavirus disease 2019 (COVID-19) in intensive care.

**Design:**

Outbreak analysis to assess for a transmission risk area (TRA) conducted after identification of potential CPE outbreak within shared room spaces in intensive care.

**Setting:**

Analysis conducted within a 24-bed single-room model intensive-care department within a level-3 tertiary center public hospital in regional Victoria, Australia.

**Patients:**

3 patients, with severe COVID-19 admitted to intensive care over a 3-month period with shared room spaces requiring prolonged mechanical ventilation and broad-spectrum antimicrobials, identified and were managed for CPE isolated from sputum. Overlap carbapenemase genes were identified among different organisms raising suspicion of transmitted resistance genes. A subsequent case managed for severe community-acquired pneumonia isolated CPE 3 months beyond these cases.

**Methods:**

Outbreak analysis via weekly cross-sectional point prevalence screening of fecal samples or rectal swabs for CPE from patients admitted to the intensive-care department over a 4-week period.

**Results:**

34 patients were included in the analysis with 51 tests for CPE screening conducted. No further cases of CPE were identified. Statewide Infection Surveillance team and the Department of Health and Human Services did not find the cases to derive from a TRA. No further action including environmental screening was indicated.

**Conclusions:**

These cases highlight the independent acquisition of CPE genes in patients with severe COVID-19 and antimicrobial selective pressures resulting in significant morbidity and mortality. Increasing awareness, robust antimicrobial stewardship, and infection prevention measures could reduce pressures driving CPE resistance mutations and the risk of CPE transmission.

## Introduction

Carbapenemase-producing Enterobacterales (CPE) are gram-negative enteric flora with acquired resistance to carbapenem antibiotics, posing diagnostic and management challenges. Infections caused by CPE are associated with significant treatment failure resulting in increased morbidity and mortality associated with infections.^1^ Nosocomial CPE infection poses unique challenges to infection control including outbreak identification and management and infection prevention measures.

We describe 3 patients with severe coronavirus disease 2019 (COVID-19) infection admitted to an intensive care unit over a 3-month period at *University Hospital Geelong*, *Barwon Health.* This service is a level-3 tertiary center, the largest regional healthcare provider, and the second largest extracorporeal membrane oxygenation service in Victoria, Australia. All received broad-spectrum antimicrobials with subsequent identification and management of CPE from sputum or tracheal samples. A subsequent fourth patient, identified 3 months after the original cases, was managed for severe community-acquired pneumonia (CAP) requiring intubation and isolated CPE.

Cases were managed within the intensive care unit over shared room spaces at different time points, raising suspicion of a transmitted resistance between patients and prompting an outbreak investigation. Given the shared environmental space, the ability of CPE organisms to transmit resistance genes via plasmids, and strong antimicrobial selective pressures secondary to broad-spectrum antimicrobial use—it is hypothesized that transmission of acquired resistance mutations may have been responsible for an outbreak within the intensive-care department.^2^

## Methods

As per the VICNISS Healthcare Associated Infection Surveillance Coordinating Centre and Department of Health and Human Services (DHHS) Victoria, weekly point prevalence screening within the intensive-care department was implemented aiming to identify a source of outbreak through a Transmission Risk Area (TRA)—defined as space (geographic area or ward) where local transmission of an infection has occurred.

Via weekly cross-sectional analysis, point prevalence screening for identification of CPE was conducted within the intensive-care department between February 16 and March 9, 2022. This process implemented weekly fecal sample or rectal swab testing for CPE identification over a total 4-week period. No sampling of other body sites was included in the analysis. All patients admitted to the intensive-care department on each day of screening were included in this analysis.

Though case 4 identified a similar organism and resistance profile, as there was a significant difference in periods between the original cases (greater than 6 weeks), both VICNISS and DHHS did not recommend further analysis.

Laboratory methods included CPE screening samples cultured on a bi-plate format Brilliance ESBL/CRE chromogenic agar (Oxoid, Basingstoke, UK) aerobically at 37°C for 48 hours. Colonies were sampled and identification was performed by matrix-assisted laser desorption/ionization time-of-flight (MALDI-TOF; Bruker, Billerica, MA). Meropenem resistance testing was conducted via VITEK® 2 microbial identification and antibiotic susceptibility testing (bioMerieux, Marcy l’Etoile, France) and interpreted using Clinical and Laboratory Standards Institute standards and guidelines.

Carbapenemase production was assessed via the carbapenemase inactivation method (CIM), with a positive CIM test indicating carbapenemase detection. All positive CPE samples were sent to the Microbiological Diagnostic Unit, University of Melbourne, for confirmatory molecular identification and antimicrobial susceptibility testing (Broth Microdilution).

### Description of outbreak

**Figure 1.**
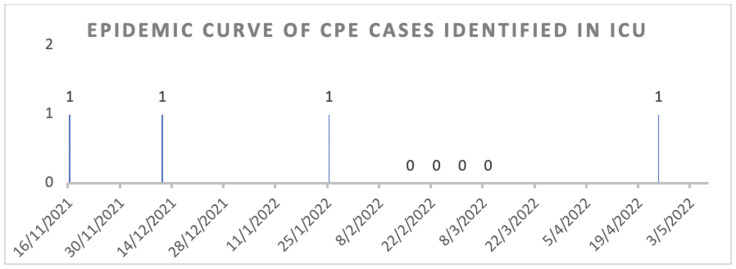
Epidemic curve identifying cases of carbapenemase-producing Enterobacterales (CPE) in the intensive care unit (ICU) between November 2021 and April 2022 and results of weekly CPE screening in patients within the ICU conducted between February to March 2022.

Three cases of CPE identified from sputum samples of patients managed for severe COVID-19 infection in an intensive-care department over a 3-month period raised suspicion for transmission of carbapenem resistance genes and triggered outbreak surveillance (Figure 1: Epidemic curve). A subsequent fourth case of CPE beyond surveillance was not deemed significant.

**Figure 2.**
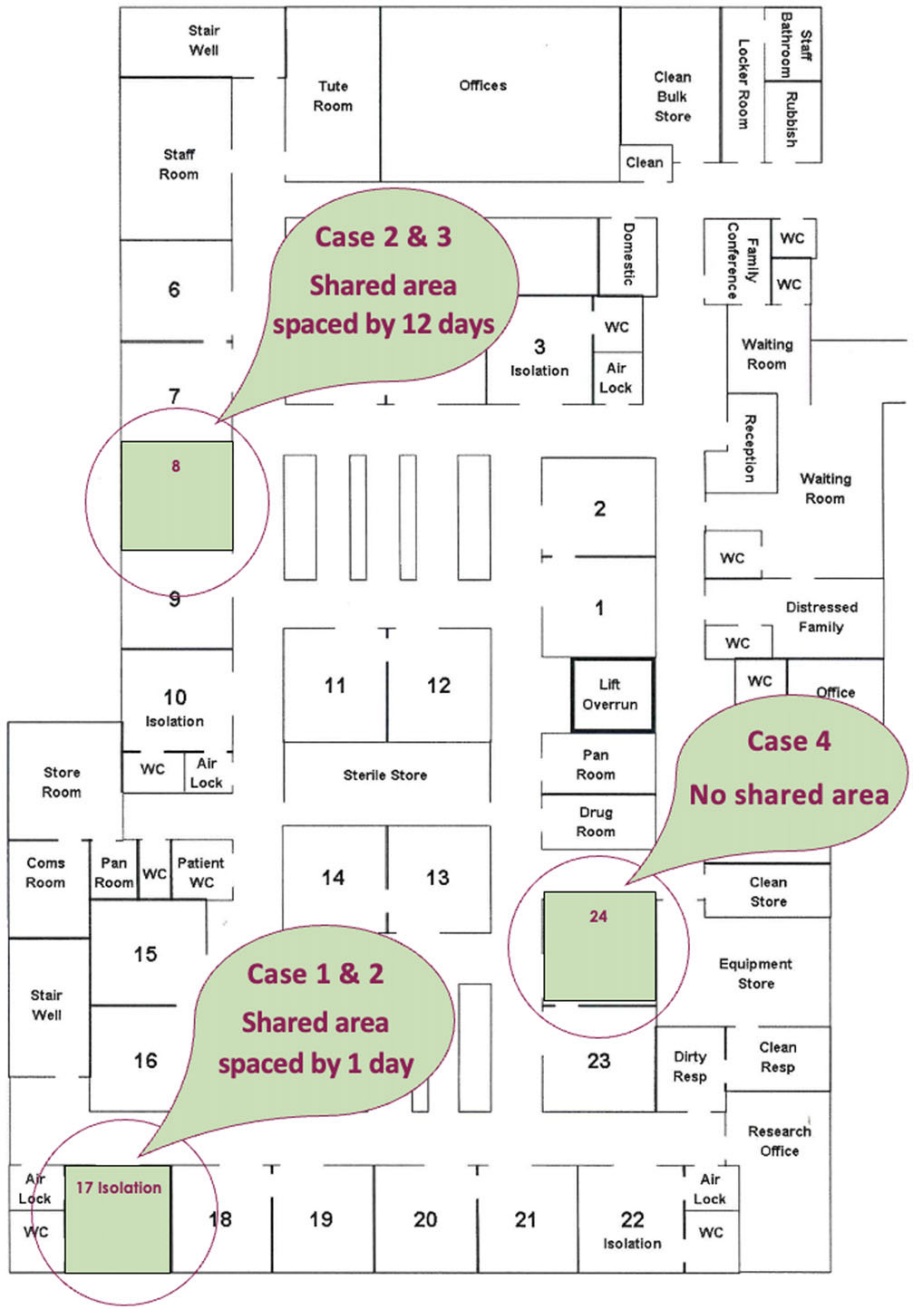
Floor plan of intensive-care department at Barwon Health, University Hospital Geelong, depicting the shared room spaces by identified cases of carbapenemase-producing Enterobacterales.

This intensive-care department within the hospital is a 24-bed ward with a single-room model of care with 1 nurse allocated per patient (Figure 2: Floor plan).

**Case 1** is a 64-year-old man with a history of dilated cardiomyopathy, atrial fibrillation, type-2 diabetes mellitus (T2DM), hypertension, and active smoking and no history of COVID-19 vaccination. He presented in October 2021 with severe COVID-19 infection requiring intensive-care admission and intubation on day 2. Management included corticosteroids and baricitinib for COVID-19 infection and empirical piperacillin/tazobactam for presumed concurrent severe CAP. On day 20, wean to tracheostomy care was made. The intensive-care stay was complicated by end-stage kidney injury requiring renal replacement therapy, arrhythmia, critical illness myopathy, and multiple infectious complications including *Staphylococcus epidermidis* line-related infection requiring vancomycin therapy for 1 week, severe *Clostridioides difficile* infection requiring intravenous metronidazole and oral vancomycin for 10 days and a repeated course of vancomycin for *C. difficile* recurrence within 2 weeks. The patient was managed for ventilator-associated pneumonia within 30 days of admission with a sensitive *Pseudomonas aeruginosa* isolated on a sputum sample with piperacillin/tazobactam for 7 days.

On day 39, subsequent sputum sampling revealed persistent Pseudomonas growth alongside isolation of a carbapenemase-producing *Escherichia coli* with the detection of an OXA-48-like resistance gene (OXA-244 allele, multi-locus sequence typing [MLST] type 10) on further testing. Initially, ciprofloxacin was commenced based on the sensitivity profile; however, further respiratory deterioration prompted repeated sputum samples revealing the CPE *E. coli* had acquired fluoroquinolone resistance (Table 1: Susceptibility). A combination of ceftazidime/avibactam and amikacin was commenced for 14 days with respiratory recovery and decannulation on day 73 with subsequent discharge from intensive care on day 75. The patient died on day 77 from a presumed aspiration event and subsequent respiratory arrest.

**Table 1.** Antimicrobial susceptibility testing of carbapenemase-producing Enterobacterales organisms identified in outbreak cases in intensive care at Barwon Health, University Hospital Geelong

Antibiotic	Case 1 OXA-48 like *E. coli*	Case 2 IMP-4, MCR-9, ACT-37, SHV-12 *E. cloacae*	Case 3 IMP-4, MCR-9 *E. coli*	Case 4 IMP-4, MCR-9 *E. cloacae*
	MIC (mg/L), interpretation			
Amikacin	<2, S	4, S	8, S	<2, S
Aztreonam	>32, R	>32, R	32, R	>32, R
Cefiderocol	1, S	2, S	0.25, S	N/A
Ceftazidime-avibactam	0.5, S	>16, R	>16, R	>16, R
Ciprofloxacin	<0.25, S	>2, R	2, R	1, R
Colistin	<0.5, S	<0.5, S	<0.5, S	<0.5, S
Meropenem-vaborbactam	16, R	8, S	1, S	0.25, S

Note. MIC, minimal inhibitory concentration; S, susceptible; R, resistant.

**Case 2** is a 78-year-old man with a history of hypertension, nonsmoker, and 2 previous AstraZeneca™ vaccines for COVID-19. He presented in late November 2021 with dyspnea with transition to intensive care for intubation for progressive respiratory distress on day 3. He was managed with dexamethasone, remdesivir, and baricitinib for severe COVID-19 infection and randomized to moxifloxacin as part of a clinical trial for CAP.

On day 12, a carbapenemase-producing *Enterobacter cloacae* was identified from purulent sputum; however; in the absence of a clinical syndrome consistent with pneumonia, this pathogen was initially not treated. This isolate harbored resistance genes IMP-4, MCR-9, ACT-37, and SHV-12 (MLST type 269) with susceptibility profile summarized in Table 1.

On day 18, a transition to tracheostomy was made. Given the repeated isolation of CPE on sputum samples over 4 days and the high risks for respiratory deterioration, a decision was made to commence targeted CPE treatment with a combination of amikacin and ceftazidime/avibactam for a total of 10 days. The patient was decannulated on day 26 and discharged from hospital care on day 42.

**Case 3** is a 67-year-old man with a history of T2DM, hypertension, obesity, and active smoking and no history of COVID-19 vaccination. He presented in early January 2022 with dyspnea secondary to COVID-19 requiring intubation. He was commenced on ceftriaxone, azithromycin, and flucloxacillin for isolation of *E. coli* (amoxicillin resistant) and methicillin-sensitive *Staphylococcus aureus* on sputum sample on day 1. On day 1, the sputum sample isolated *E. coli* with an increasingly resistant pattern (resistant ampicillin, third-generation cephalosporins, and sensitive gentamicin and ciprofloxacin). The transition was made to intravenous ciprofloxacin for *E. coli* and cefazolin for *S. aureus* to reduce the risk of renal toxicity given severe acute kidney injury requiring continuous renal replacement therapy. Antimicrobials were ceased on 13 with the transition to tracheostomy on day 14.

On day 19, the patient developed respiratory and hemodynamic compromise requiring inotropic support. Sputum sample isolated *E. coli*, which prompted the broadening of antimicrobial coverage to meropenem. Within 24 hours the laboratory confirmed a positive CIM test, consistent with carbapenemase production. The reference laboratory confirmed CPE was found to harbor the IMP resistance gene and alleles IMP-4 and MCR-9 (MLST type 69). Given ongoing deterioration, a transition to ceftazidime/avibactam and aztreonam was made prior to susceptibility confirmation, which subsequently revealed resistance (Table 1: Susceptibility). Despite use for 7 days, clinical recovery was achieved with the patient decannulated on day 38.

On day 49, *Enterococcus faecalis* was identified in blood cultures with a urinary focus managed with intravenous ampicillin, concurrent respiratory sample again isolated the CPE *E. coli*; however, given respiratory stability, therapy was not commenced. A discharge to rehab care was made on day 56 with discharge home on day 68.

**Case 4** is a 65-year-old male with a history of ischemic heart disease, chronic obstructive pulmonary disease, active smoking, and penicillin allergy and no history of COVID-19 vaccination who presented with out-of-hospital cardiac arrest requiring percutaneous coronary intervention in March 2022. He presented within 2 weeks with cough and dyspnea and was found to have type-2 respiratory failure due to left lower lobe pneumonia requiring inotropic supports, intubation, and admission to the intensive care unit on day 1. He was commenced on vancomycin, cefepime, and azithromycin for severe CAP with early transition to intravenous ceftriaxone and azithromycin for 10 days.

He failed extubation on day 5 due to progressive respiratory failure and required tracheostomy on day 14, complicated by tracheal bleeding requiring extraction of obstructive clots in theater. On day 19, increasing sputum production led to the isolation of *Enterobacter cloacae* found to harbor resistance genes IMP-4 and MCR-9 (MLST type 269) with susceptibilities summarized in Table 1. This was managed with amikacin and aztreonam and transitioned to amikacin based on sensitivity profile for 10 days until day 29. The patient underwent decannulation on 30; however, was transitioned to end-of-life care due to recurrent aspiration and respiratory decline. He died on day 52.

## Results and public health response

VICNISS and DHHS incident management teams were involved in decision-making to aid outbreak management. Recommendations were made to initiate point prevalence analysis to aid assessment of identification of potential TRA. Further actions if a potential TRA was identified were to escalate to staff screening and environmental surveillance. During this period, increased environmental cleaning was incorporated within the unit as a proactive measure in the case of a potential environmental source of the CPE outbreak.

A total of 43 patients occupied a bed space within the intensive-care department over the defined 4-week period of outbreak analysis. Of these, 34 (79.1%) underwent CPE surveillance testing. Nine (20.9%) patients within the department were eligible for analysis on days of testing, however did not undergo screening due to being discharged prior to testing occurring on designated days and were not included within the surveillance analysis.

Over the 4-week outbreak analysis period, a total of 51 CPE screening tests were performed. Of these, 17 (31.5%) tests were repeated screening tests in patients who had been tested in the preceding week(s). There was a total of 12 isolated incidents of patients not undergoing testing due to early discharge from the unit—6 cases in week 2 and 6 in week 3. Of these 12 incidents, 3 were in patients who had tested negative within the preceding week.

Over the 4-week outbreak analysis period, surveillance screening of a total of 34 patients through 51 total CPE tests did not identify any further cases of CPE. Incidental discovery of 2 cases of ESBL organisms on fecal samples was made—these were deemed not significant in our analysis given different resistance mechanisms.

The site was not deemed to be a TRA implying local transmission between patients was not thought to have occurred. As a result of this analysis, no further staff screening, environmental analysis, or cleaning processes were warranted nor any further analysis of infection control methods within the intensive care unit.

The subsequent discovery of case 4 with similar organism and resistance profiling beyond surveillance, as directed by VICNISS and DHHS, was classified as unrelated given the time interval between cases. Further investigation was not deemed relevant.

## Discussion

The CPE cases described in this work represent the first reports of acquired CPE within *University Hospital Geelong*. Our outbreak analysis did not identify a high-risk transmission area; however, suspicion remains for possible resistance gene transmission among the patient cases.

The mechanism of carbapenem resistance is achieved through enzymatic production via the expression of carbapenemase genes. These genes reside within highly mobile elements of genetic material with the potential for transmissibility among isolates and between persons. In our study, cases 2 and 3, who shared a room space, harbored similar carbapenemase resistance gene IMP-4.

IMP group mutations are classified as metallo-beta-lactamases (MBLs) and belong to the class B beta-lactamase group. A variety of MBL genes have been associated with carbapenem resistance including IMP-1, IMP-7, IMP-9, IMP-26, VIM-2, and VIM-6.^3,4^ These genes encode zinc-dependent enzymes that function via beta-lactam hydrolysis inferring resistance to beta-lactamase inhibitors and hence resistance to beta-lactams antimicrobial class including carbapenems.^5^

Acquired MBLs are encoded within integrons carried by plasmids with the ability for horizontal transmissibility among genera or species.^2^ Despite no further CPE or resistance gene acquisition identified within this analysis, it is worth considering if the implicated IMP-4 mutation carried by the *Enterobacter cloacae* in case 2 was transmitted to the *E. coli* in case 3 given a shared room space.

CPE resistance genes or organism transmission is described within hospital settings among patients—commonly via spread from healthcare worker hand carriage, shared environmental surfaces, or shared equipment.^6^ Reduction of spread through adherence to adequate infection control techniques is pivotal. These techniques include adopting contact precautions where appropriate and ensuring hand hygiene, environmental cleaning, and reprocessing of medical equipment are practiced.^7^

Host factors including impaired immunity and use of broad-spectrum antimicrobials are known risk factors for CPE acquisition.^8,9^ Our cases represent a cohort of comorbid patients who spent a prolonged time in intensive care, required mechanical ventilation, were exposed to broad-spectrum antimicrobials, and were immunosuppressed through COVID-19 therapies. These risk factors may confer a significant and additive risk for the independent acquisition of CPE infection.^10^

The contribution of selective pressures leading to resistance is demonstrated among the cases—each case initially isolates sensitive organisms early within their admission and subsequently identifies resistant organisms including CPEs as they become exposed to cumulative risks for severe and resistant infection. Awareness of pressures driving CPE resistance mutations allows the opportunity to adopt transmission reduction methods.

A coordinated public health response aims to adopt early detection of CPE infections within high-risk populations to institute targeted therapies while also maximizing preventative measures. A strategic road map to control CPE outbreaks can be promoted across hospitals at a national scale.^11^

Antimicrobial stewardship (AMS) programs have an impact on addressing CPE outbreaks in high-risk settings.^12,13^ The incorporation of AMS programs in consult with infectious diseases was successful in reducing CPE and mortality mediated by CPE.^14^ Our study reinforces the importance of AMS programs to identify susceptibility profiles of local isolates and provide an opportunity to audit antimicrobial prescribing behaviors, review compliance, and invite review of guidelines and practice. AMS programs in combination with targeted infection control methods and outbreak analysis teams will continue to play an important role in the prevention and management of CPEs.

This outbreak report represents the apparent independent acquisition of CPE-related resistance genes among patients within a shared environment in the setting of severe COVID-19 or respiratory infection and selective pressures by broad-spectrum antimicrobial use. The significance of a shared environmental space among these cases remains unclear but may have contributed to the transmission of CPE resistance genes.

These clinical cases highlight the importance of awareness of multi-drug-resistant organisms due to the selection pressures in severely unwell patients in intensive care with COVID-19 infection or pneumonia, the importance of AMS programs to reduce inappropriate broad-spectrum antimicrobial use, and the need for transmission reduction methods via infection control measures.
